# RobustTree: An adaptive, robust PCA algorithm for embedded tree structure recovery from single-cell sequencing data

**DOI:** 10.3389/fgene.2023.1110899

**Published:** 2023-03-08

**Authors:** Ziwei Chen, Bingwei Zhang, Fuzhou Gong, Lin Wan, Liang Ma

**Affiliations:** ^1^ Department of Systems Biology, Columbia University Irving Medical Center, New York, NY, United States; ^2^ Academy of Mathematics and Systems Science, Chinese Academy of Sciences, Beijing, China; ^3^ School of Mathematical Sciences, University of Chinese Academy of Sciences, Beijing, China; ^4^ Institute of Zoology, Chinese Academy of Sciences, Beijing, China

**Keywords:** single-cell sequencing, robust principal component analysis, data denoising, clustering, tree structure reconstruction

## Abstract

Robust Principal Component Analysis (RPCA) offers a powerful tool for recovering a low-rank matrix from highly corrupted data, with growing applications in computational biology. Biological processes commonly form intrinsic hierarchical structures, such as tree structures of cell development trajectories and tumor evolutionary history. The rapid development of single-cell sequencing (SCS) technology calls for the recovery of embedded tree structures from noisy and heterogeneous SCS data. In this study, we propose RobustTree, a unified framework to reconstruct the inherent topological structure underlying high-dimensional data with noise. By extending RPCA to handle tree structure optimization, RobustTree leverages data denoising, clustering, and tree structure reconstruction. It solves the tree optimization problem with an adaptive parameter selection scheme that we proposed. In addition to recovering real datasets, RobustTree can reconstruct continuous topological structure and discrete-state topological structure of underlying SCS data. We apply RobustTree on multiple synthetic and real datasets and demonstrate its high accuracy and robustness when analyzing high-noise SCS data with embedded complex structures. The code is available at https://github.com/ucasdp/RobustTree.

## 1 Introduction

Cell fate decisions and tumorigenesis are complex biological processes that experience many state transitions, such as cell differentiation and somatic cell evolution. The rapid development of single-cell sequencing (SCS) technology makes it possible to unveil the dynamics of such biological processes. However, the limited genetic content of a single cell and the stochastic nature of sequencing techniques can result in high rates of gene dropout and various sequencing errors, leading to noisy SCS data (Gawad et al., 2016; Chen et al., 2022; Wen et al., 2022). It poses additional challenges to reconstruct the potential hierarchical topological structure or dynamics of cells from such noisy high-dimensional SCS data.

In recent years, recovering intrinsic structure from high-dimensional data has become a central topic in the data science and machine learning community. Pioneering works generally seek to perform data dimensionality reduction or associate data with certain structured objects (Mao et al., 2016). For example, principal curve (Hastie and Stuetzle, 1989) and its successors (Tibshirani, 1992; Bishop et al., 1998; Kégl et al., 2000; Smola et al., 2001; Sandilya and Kulkarni, 2002; Olivas et al., 2009), fits/maps an infinitely differentiable curve with a finite length to pass through the middle of data. However, these methods cannot handle self-intersection data. To address this gap, principal graph method uses a collection of piecewise smooth curves to approximate the data structures with self-intersection (Kégl and Krzyzak, 2002). Topological data analysis, which performs graph representation of high-dimensional datasets, provides another way to handle self-intersection structure (Carlsson, 2009). Ge et al. (2011) develops an efficient topological data analysis algorithm, which uses Reeb graphs (Dey and Wang, 2011) to extract a one-dimensional skeleton from unorganized data. Another tool, Mapper (Singh et al., 2007), builds simplicial complexes to preserve certain topological structures from the original dataset, and has been applied on single-cell data to reconstruct the dynamical structures of cell states (Rizvi et al., 2017). In addition, Mao et al. (2016) proposes a principal graph and structure learning framework based on reversed graph embedding (RGE) to capture the local information of the underlying graph structure. RGE is subsequently equipped by Monocle2 (Qiu et al., 2017) for single-cell trajectory inference.

The high noise in SCS data, which is brought by either technological or/and experimental issues, could possibly affect downstream clustering or distort the reconstruction of intrinsic structures. A number of computational methods have been proposed to retrieve lost and corrupted information from SCS data (Chen Z. et al., 2020; Patruno et al., 2021). Among them, methods based on matrix decomposition are computationally more efficient, especially extensions of robust principle component analysis (RPCA) algorithms. RPCA is an efficient low-rank matrix decomposition method for recovering low-dimensional subspace from corrupted data (Lin et al., 2010; Candes et al., 2011; Hsu et al., 2011; Vidal et al., 2016), which has been applied to denoise either DNA or RNA profiles of SCS data (Chen C. et al., 2020; Chen Z. et al., 2020; Su et al., 2022). Since the assayed cells often come from a few states in cell development or clones in tumor, and cells of same state or clone have similar or identical expression or genomic profile. In addition, noise in observed SCS data generally introduced by technologies can be random and sparse (Chen Z. et al., 2020; Su et al., 2022). Therefore, SCS data well suits the low-rank plus sparse matrices assumptions of RPCA.

In this study, we propose a unified framework, termed RobustTree, to reconstruct the inherent topological structure underlying high-dimensional data with high noise. The framework involves a matrix decomposition process that recovers the latent data points in a low-rank space. And these data points are used directly to reconstruct a tree to represent the inherent single-cell evolutionary trajectory. We also introduce a discriminative and compact feature representation for clustering problems with an assumption that the cluster centers should be close to each other when connected on the learned tree structure, otherwise they should be distant (Mao et al., 2015). More specifically, the optimization objective function of the framework consists of the following three basic components, including 1) denoising using robust principal component analysis (RPCA) and extended RPCA method; 2) performing data clustering with a soft assignment strategy; 3) reconstructing the minimum spanning tree (MST) among cluster centers as the potential topological structure. RobustTree leverages data denoising, clustering, and tree structure reconstruction and solves the tree optimization problem with an adaptive parameter selection scheme. Based on adaptive trade-off parameters, RobustTree not only can reconstruct continuous topological structure, e.g., cell development trajectory based on single-cell RNA sequencing gene expression data, but also display discrete-state topological structure, e.g., tumor evolution history based on single-cell DNA sequencing genetic variant data, including single-cell single nucleotide variation (scSNV) data and single-cell copy number alteration (scCNA) data. By using multiple simulated and real datasets, we demonstrate that RobustTree is accurate and robust on high-noise data with complex structures.

## 2 Materials and methods

Let $X_{M\times N}\in X$ represent an observed noisy SCS data matrix where the rows represent data points, such as cells, and columns represent features, such as genes or mutations. We consider recovering a latent low-rank matrix *A*_*M*×*N*_ corresponding to each *X*_*M*×*N*_. In the recovered low-dimensional space, the same, or similar, data points are aggregated into *K* clusters, and we reconstruct a tree-like structure T at the cluster level to represent the true topology of the data.

In the following, we introduce three components of the proposed framework, including 1) RPCA and extended RPCA algorithms (Section 2.1), which are used to recover low-rank subspace from data matrix with corrupted and/or missing entries, 2) data clustering (Section 2.2) and 3) the minimum spanning tree (MST) optimization problem (Section 2.3). Finally, we describe our RobustTree framework, which is a low-rank matrix recovery framework coupled with tree structure optimization (Section 2.4).

### 2.1 RPCA and extended RPCA

#### 2.1.1 RPCA

The celebrated dimensionality reduction method, PCA (Vidal et al., 2005), which assumes that noise follows a Gaussian distribution, is unrobust to in-sample outliers. As a consequence, the robust PCA (RPCA) (Lin et al., 2010; Candes et al., 2011; Hsu et al., 2011; Vidal et al., 2016) emerges to recover the potential low-rank matrix from data with sharp and sparse noise.

Assume that the observed data matrix *X*_*M*×*N*_ is generated by the sum of two matrices *X* = *A*_0_ + *E*_0_, where *A*_0_ is a low-rank matrix, and *E*_0_ represents the intra-sample outliers affected by random sparse noise, then the RPCA problem can be formulated as:

$$\underset{A,E}{\min}rank\left(A\right)+\lambda \|E{\|}_{0},\;s.t.\;A+E=X,$$ (1)

where ‖ ⋅‖_0_ denotes 0-norm of the matrix (i.e., the number of non-zero entries in the matrix), and *λ* is a trade-off parameter. However, solving Problem Eq. 1 is generally NP-hard (Vidal et al., 2005; Chen Z. et al., 2020). Therefore, in order to reduce the above computation burden, Problem Eq. 1 can be convexly relaxed as

$$\underset{A,E}{\min}\|A{\|}_{*}+\lambda \|E{\|}_{1},\;s.t.\;A+E=X,$$ (2)

where ‖ ⋅‖_*_ and ‖⋅‖_1_ represent the nuclear norm and the *ℓ*_1_ norm of the matrix, respectively. We refer to Problem Eq. 2 as a relaxed version of RPCA, which can be efficiently solved by augmented Lagrange multipliers (ALM) (Lin et al., 2010).

#### 2.1.2 Extended RPCA

In practice, dropout/missing events can occur frequently in the observed matrix, except for corrupted entries. In order to model the missing entries, we first define a linear mapping $P_{\Phi }\left(\cdot \right):R^{M\times N}\to R^{M\times N}$ , which maps the missing entries to 0 and keeps the observed entries, i.e., ${\left[P_{\Phi }\left(X\right)\right]}_{i,j}=X_{i,j}$ if (*i*, *j*) ∈ Φ, and ${\left[P_{\Phi }\left(X\right)\right]}_{i,j}=0$ otherwise. Then, RPCA problem can be extended to the following version (Wright et al., 2013; Shang et al., 2014; Vidal et al., 2016; Chen Z. et al., 2020):

$$\underset{A,E}{\min}\|A{\|}_{*}+\lambda \|E{\|}_{1},\;\text{s.t.}\;P_{\Phi }\left(A+E\right)=P_{\Phi }\left(X\right),$$ (3)

which aims to decompose *X* into a low-rank matrix *A* and sparse component *E* based only on the observed data *P*_Φ_(*X*). The optimization Problem Eq. 3 is shown to be equivalent to solving the following constrained optimization problem (Shang et al., 2014):

$$\underset{A,E}{\min}\|A{\|}_{*}+\lambda \|P_{\Phi }\left(E\right){\|}_{1}\;\text{s.t.}\;A+E=X,$$ (4)

which can also be solved by the ALM algorithm (Shang et al., 2014; Chen Z. et al., 2020). It is worth noting that when Φ is the index set of all entries in *X*, Problem Eq. 4 can be transformed into Problem Eq. 2; that is, Problem Eq. 2 can be regarded as a special case of Problem Eq. 4. Thus, we can solve these problems in a unified form provided by Problem Eq. 4.

### 2.2 Data clustering

The second component of our proposed framework is clustering of the recovered low-rank matrix. Since RPCA-based recovery of low-rank matrices may not fully guarantee an error-free state, we cannot obtain cluster centers by simply merging rows with the same features in the low-rank matrix *A* (Chen Z. et al., 2020). Assume there exist *K* clusters in the data points. Then we denote *C*_*k*_ as the *k*th cluster centroid of *A* (*k* ∈ {1, …, *K*}). We minimize the following quantization error (Smola et al., 2001) to get the optimal cluster centroids:

$$\overset{M}{\underset{i=1}{\sum }}\underset{k=1,\ldots ,K}{\min}\|A_{i}-C_{k}{\|}_{2}^{2}.$$ (5)

When *K* < *M*, we introduce an indicator matrix Δ ∈ [0,1]^*M*×*K*^, where the (*i*, *k*)th element *δ*_*i,k*_ = 1 indicates that data point *A*_*i*_ is assigned to the *k*th cluster, and *δ*_*i,k*_ = 0 otherwise. Then, we get the equivalent optimization objective as follows:

$$\overset{M}{\underset{i=1}{\sum }}\overset{K}{\underset{k=1}{\sum }}\delta_{i,k}\|A_{i}-C_{k}{\|}_{2}^{2},$$ (6)

where $\sum_{k=1}^{K}\delta_{i\mathrm{,k}}=1$ and *δ*_*i,k*_ ∈ {0, 1} for *∀i* ∈ {1, …, *M*}. This is the same optimization objective as *K*-means clustering. However, when *K* is relatively large, *K*-means with minimization of optimization Problem Eq. 6 might generate empty clusters. To avoid this, we introduce a right stochastic matrix *R*, where $\sum_{k=1}^{K}r_{i,k}=1,\forall i=1,\ldots ,M$ . When the obtained *R* is an integer solution, this variant is equivalent to the above representation with the indicator matrix Δ. Subsequently, we follow (Mao et al., 2016) and employ the following soft assignment strategy by adding negative entropy regularization, as:

$$\overset{M}{\underset{i=1}{\sum }}\overset{K}{\underset{k=1}{\sum }}r_{i,k}\left(\|A_{i}-C_{k}{\|}_{2}^{2}+\sigma \log r_{i,k}\right),$$ (7)

where *σ* > 0 is a regularization parameter. When *R* ∈ [0,1]^*M*×*K*^ is a left stochastic matrix with each column summing to one, optimization Problem Eq. 7 is equivalent to mean shift clustering, and when *R* ∈ [0,1]^*M*×*K*^ is a right stochastic matrix with each row summing to one, optimization Problem Eq. 7 is equivalent to the Gaussian mixture model with uniform weights (Mao et al., 2016).

### 2.3 Minimum spanning tree (MST)

MST is a common graphical representation applied in the reconstruction of dynamic biological processes, such as cell developmental trajectory reconstruction and tumor evolutionary history recovery (Gawad et al., 2014; Yuan et al., 2015; Ross and Markowetz, 2016; Qiu et al., 2017; Chen et al., 2019; Chen Z. et al., 2020). MST characterizes lineage tracing path between different cell states or tumor clones and can explicitly reflect the process of cell development or the progression of subclones (Chen Z. et al., 2020).

We follow the MST optimization scheme proposed by Mao et al. (2015). Let G=(V,E) denote a connected undirected graph with weight *W*, where V={1,…,K} is a set of vertices, E is a set of edges and the entry *w*_*i*,*j*_ in *W* represents the weight associated with the edge $\left(V_{i},V_{j}\right)\in E,\forall i,j\in V$ . Then we define a tree $T=\left(V,E_{T}\right)$ on the graph G that connects all vertices with minimum total weight, where $E_{T}$ contains the edge set of tree T . In order to represent and learn a tree, we consider {*b*_*i*,*j*_} as binary variables; that is,

$$b_{i,j}=\left\{\begin{array}{cc} 1 & if\;\left(V_{i},V_{j}\right)\in E_{T};\\ 0 & otherwise. \end{array}\right.$$

Let *B* = [*b*_*i*,*j*_] ∈ {0,1}^*K*×*K*^; Then the integer linear programming formulation of MST can be represented as follows (Mao et al., 2015):

$$\begin{array}{cc} & \underset{B\in B}{\min}\underset{i,j}{\sum }b_{i,j}w_{i,j},\\ s.t.B & =\left\{B\in {\left\{0,1\right\}}^{K\times K}\right\}⋂B^{\prime },\;\\ B^{\prime } & =\left\{B=B^{T}\right\}⋂\left\{\frac{1}{2}\underset{i,j}{\sum }b_{i,j}=|V|-1\right\}⋂\left\{\frac{1}{2}\underset{V_{i}\in S,V_{j}\in S}{\sum }b_{i,j}=|S|-1\right\},\forall S\subseteq V. \end{array}$$ (8)

Worth noting that there are three constraints applied to set $B^{\prime }$ , including 1) connection symmetry as an undirected graph; 2) restriction of spanning trees containing |V|−1 edges; and 3) acyclic and connectivity of a spanning tree. Instead of solving such an integer programming problem directly, which is very difficult, we can relax the problem as

$$\underset{B\in B}{\min}\underset{i,j}{\sum }b_{i,j}w_{i,j},$$ (9)

where *b*_*i*,*j*_ ≥ 0, the set of linear constraints is given by $B=\left\{B\geq 0\right\}⋂B^{\prime }$ (Mao et al., 2015). Then Problem Eq. 9 can be solved by Kruskal’s algorithm (Kruskal, 1956; Cormen et al., 2022).

### 2.4 Low-rank matrix recovery coupled with tree structure optimization

Based on the three building blocks described in Sections 2.1, Sections 2.2, and Sections 2.3, we are ready to formulate our unified framework to learn the latent topological structure hidden underneath noisy SCS data. We use the alternating direction multiplier method (ADMM) to solve the proposed optimization objective function by simultaneously recovering real data points and learning a tree-like structure with guaranteed convergence.

#### 2.4.1 RobustTree framework

Given observed input SCS data *X*_*M*×*N*_, our goal is to reveal the underlying tree structure that generates *X*. Since the observed data may be corrupted by noise, it is not appropriate to recover the underlying topology directly from the observation matrix. Instead, to unveil the underlying structure, we assume that a latent low-rank matrix *A*_*M*×*N*_ can be recovered from *X*_*M*×*N*_, and we focus on learning a tree structure $T=\left(V,E_{T}\right)$ on the cluster level of *A*. Let *C*_*K*×*N*_ indicate the cluster centers of data points in *A*, and *B*_*K*×*K*_ represent the adjacency matrix of vertices V in graph T ; then, the optimization problem with respect to variables {*A*, *E*, *C*, *B*, *R*} is formulated as:

$$\begin{array}{cc} & \underset{A,E,B,C,R}{\min}\;\|A{\|}_{*}+\lambda \|P_{\Phi }\left(E\right){\|}_{1}+\frac{\theta }{2}\underset{k,k^{\prime }}{\sum }b_{k,k^{\prime }}\|C_{k}-C_{k^{\prime }}{\|}_{F}^{2}+\gamma \left[\overset{K}{\underset{k=1}{\sum }}\overset{m}{\underset{i=1}{\sum }}r_{i,k}\|A_{i}-C_{k}{\|}^{2}+\sigma \Omega \left(R\right)\right]\\ & \;s.t.A+E=X,B\in B,\overset{K}{\underset{k=1}{\sum }}r_{i,k}=1,r_{i,k}\geq 0,\forall i,\forall k, \end{array}$$ (10)

where *λ*, *θ*, *γ* and *σ* are trade-off parameters, *r*_*i*,*k*_ indicates the (*i*, *k*)th entry in matrix $R\in R^{M\times K}$ , $\Omega \left(R\right)=\sum_{i=1}^{M}\sum_{k=1}^{K}r_{i,k}\log r_{i,k}$ represents negative entropy regularization, and the definition of B is detailed in optimization Problem Eq. 8.

#### 2.4.2 RobustTree framework optimization algorithm

We solve optimization Problem Eq. 10 by alternating direction multiplier method (ADMM). We divide variables into two disjoint groups as {*A*, *E*, *C*} and {*B*, *R*}, and then solve each subproblem iteratively until convergence is achieved. We show details of the two following subproblems below.

• Fix {*B*, *R*} and update {*A*, *C*, *E*}

When fixing {*B*, *R*}, Problem Eq. 10 can be simplified into the following subproblem:

$$\begin{array}{c} \underset{A,E,C}{\min}\;\|A{\|}_{*}+\lambda \|P_{\Phi }\left(E\right){\|}_{1}+\frac{\theta }{2}\underset{k,k^{\prime }}{\sum }b_{k,k^{\prime }}\|C_{k}-C_{k^{\prime }}{\|}_{F}^{2}+\gamma \overset{K}{\underset{k=1}{\sum }}\overset{m}{\underset{i=1}{\sum }}r_{i,k}\|A_{i}-C_{k}{\|}^{2}\\ \;s.t.A+E=X. \end{array}$$ (11)

The corresponding augmented Lagrange function is:

$$\begin{array}{cc} L\left(A,C,E\right) & =\|A{\|}_{*}+\lambda \|P_{\Phi }\left(E\right){\|}_{1}+<\Lambda ,X-A-E>+\frac{\mu }{2}\|X-A-E{\|}_{F}^{2}\\ & \;+\frac{\theta }{2}\underset{k,k^{\prime }}{\sum }b_{k,k^{\prime }}\|C_{k}-C_{k^{\prime }}{\|}_{F}^{2}+\gamma \underset{i}{\sum }\underset{k}{\sum }r_{i,k}\left(\|A_{i}-C_{k}{\|}^{2}\right). \end{array}$$ (12)

Optimization Problem Eq. 12 can be transformed into the following form after some matrix manipulations:

$$\begin{array}{cc} L\left(A,C,E\right) & =\|A{\|}_{*}+\lambda \|P_{\Phi }\left(E\right){\|}_{1}+<\Lambda ,X-A-E>+\frac{\mu }{2}\|X-A-E{\|}_{F}^{2}\\ & +\theta trace\left(C^{T}LC\right)+\gamma \left[trace\left(A^{T}A\right)-2trace\left(R^{T}AC^{T}\right)+trace\left(C^{T}\Gamma C\right)\right]\\ & \text{where}\;L=\mathrm{diag}\left(B1_{K}\right)-B,\;\Gamma =\mathrm{diag}\left(1^{T}R\right). \end{array}$$ (13)

Updating *C*.

Let the partial derivative of L(A,C,E) with respect to *C* be zero, i.e., $\partial L\left(A,C,E\right)/\partial C=\theta LC-\gamma R^{T}A+\gamma \Gamma C=0$ ; then, we can obtain an analytical solution of *C* given by

$$C_{A}={\left(\frac{\theta }{\gamma }L+\Gamma \right)}^{-1}R^{T}A.$$

Substituting *C*_*A*_ into L(A,C,E) , we have

$$\begin{array}{cc} L\left(A,C_{A},E\right) & =\|A{\|}_{*}+\lambda \|P_{\Phi }\left(E\right){\|}_{1}+<\Lambda ,X-A-E>+\frac{\mu }{2}\|X-A-E{\|}_{F}^{2}\\ & \;+\gamma \left[trace\left(A^{T}A\right)-trace\left(A^{T}R{\left(\frac{\theta }{\gamma }L+\Gamma \right)}^{-1}R^{T}A\right)\right]. \end{array}$$ (14)

Updating *E*.

By retaining items related only to *E* in Problem Eq. 14, we have

$$\begin{array}{c} L^{\prime }\left(E\right)=\lambda \|P_{\Phi }\left(E\right){\|}_{1}+<\Lambda ,X-A-E>+\frac{\mu }{2}\|X-A-E{\|}_{F}^{2}. \end{array}$$ (15)

Solution *E* of Problem Eq. 15 can be written as (Shang et al., 2014; Chen Z. et al., 2020):

$$\begin{array}{cc} {\left[E_{k+1}\right]}_{\Phi } & ={\left[S_{\lambda \mu_{k}^{-1}}\left(X-A_{k}+\mu_{k}^{-1}\Lambda_{k}\right)\right]}_{\Phi }\\ {\left[E_{k+1}\right]}_{\Phi^{C}} & ={\left[X-A_{k}+\Lambda_{k}\right]}_{\Phi^{C}}. \end{array}$$ (16)

Updating *A*.

After removing items unrelated to *A* in Problem Eq. 14, we have

$$\begin{array}{cc} L^{\prime }\left(A\right) & =\|A{\|}_{*}+<\Lambda ,X-A-E>+\frac{\mu }{2}\|X-A-E{\|}_{F}^{2}\\ & \;+\gamma \left[trace\left(A^{T}A\right)-trace\left(A^{T}R{\left(\frac{\theta }{\gamma }L+\Gamma \right)}^{-1}R^{T}A\right)\right]. \end{array}$$ (17)

Then Problem Eq. 17 is equivalent to

$$\begin{array}{cc} L^{\prime }\left(A\right) & =\|A{\|}_{*}+<\Lambda ,X-A-E_{k+1}>+\frac{\mu }{2}\|X-A-E_{k+1}{\|}_{F}^{2}\\ & \;+\gamma \left[\|A{\|}_{F}^{2}-\|S^{T}A{\|}_{F}^{2}\right], \end{array}$$ (18)

where $S=R{\left(\frac{\theta }{\gamma }L+\Gamma \right)}^{-1/2}$ . We apply a proximity gradient algorithm to solve Problem Eq. 18. Let

$$g\left(A\right)=\|A{\|}_{*},$$

$$f\left(A\right)=-<\Lambda ,A>+\frac{\mu }{2}\|X-A-E{\|}_{F}^{2}+\gamma \left(\|A{\|}_{F}^{2}-\|S^{T}A{\|}_{F}^{2}\right),$$

then $L^{\prime }\left(A\right)=g\left(A\right)+f\left(A\right)$ . Obviously, $g\left(A\right):R^{m\times n}\to \left(-\infty ,+\infty \right)$ is a convex function, and $f\left(A\right):R^{m\times n}\to \left(-\infty ,+\infty \right)$ is a smooth convex function. Thus, $\min_{A}L^{\prime }\left(A\right)$ can be solved by a proximity gradient algorithm. Assume that the gradient of *f*(*A*) is Lipschitz continuous and that its constant is *L*_*f*_, i.e.,

$$\|\nabla f\left(X\right)-\nabla f\left(Y\right)\|\leq L_{f}\|X-Y\|,\forall X,Y\in R^{m\times n}.$$

Owing to

$$\begin{array}{cc} \nabla f\left(A\right) & =-\Lambda +\mu \left(A-\left(X-E\right)\right)+2\gamma A-2\gamma SS^{T}A\\ & =-\Lambda +\mu \left(A-\left(X-E\right)\right)+2\gamma \left(I-SS^{T}\right)A, \end{array}$$

we have

$$\begin{array}{cc} \left\|\nabla f\left(X\right)-\nabla f\left(Y\right)\right\| & =\|\mu \left(X-Y\right)+2\gamma \left(I-SS^{T}\right)\left(X-Y\right)\|\\ & =\|\left[\left(\mu +2\gamma \right)I-2\gamma SS^{T}\right]\left(X-Y\right)\|\\ & \leq \|X-Y\|\;\|\left(\mu +2\gamma \right)I-2\gamma SS^{T}\|. \end{array}$$

Then, we can set *L*_*f*_ = ‖(*μ* + 2*γ*)*I* − 2*γSS* ^*T*^‖. Considering the quadratic approximation function of $L^{\prime }\left(A\right)$ at a given point *A*_*k*_:

$$L^{\prime }\left(A,A_{k}\right)=f\left(A_{k}\right)+<\nabla f\left(A_{k}\right),A-A_{k}>+\frac{L_{f}}{2}\|A-A_{k}{\|}_{F}^{2}+g\left(A\right),$$ (19)

since formula (19) is a strong convex function, a primal solution exists for $L^{\prime }\left(A,A_{k}\right)$ , i.e.,

$$\begin{array}{cc} \arg\underset{A}{\min}\;L^{\prime }\left(A,A_{k}\right) & =\arg\underset{A}{\min}\;f\left(A_{k}\right)+<\nabla f\left(A_{k}\right),A-A_{k}>+\frac{L_{f}}{2}\|A-A_{k}{\|}_{F}^{2}+g\left(A\right)\\ & =\arg\underset{A}{\min}\;g\left(A\right)+\frac{L_{f}}{2}\|A-\left(A_{k}-\frac{1}{L_{f}}\nabla f\left(A_{k}\right)\right){\|}_{F}^{2}\\ & ={prox}_{g/L_{f}}\left(A_{k}-\frac{1}{L_{f}}\nabla f\left(A_{k}\right)\right). \end{array}$$

Let $G_{k}=A_{k}-\frac{1}{L_{f}}\nabla f\left(A_{k}\right)=A_{k}-\frac{1}{L_{f}}\left(-\Lambda +\mu \left(A_{k}-\left(X-E_{k+1}\right)\right)+2\gamma \left(I-SS^{T}\right)A_{k}\right)$ and perform the singular value decomposition on *G*_*k*_ with $G_{k}=U_{k}\Sigma V_{k}^{T},\;\;\Sigma =\mathrm{diag}{\left\{\sigma_{i}\right\}}_{1\leq i\leq r}$ . Then, $\forall 1/L_{f}>0,{prox}_{g/L_{f}}\left(G_{k}\right)=U_{k}\Sigma_{g/L_{f}}V_{k}^{T},\Sigma_{g/L_{f}}=\mathrm{diag}\left({\left\{\sigma_{i}-\frac{1}{L_{f}}\right\}}_{+}\right),{\left\{\cdot \right\}}_{+}=\max\left\{0,\cdot \right\}$ . Thus, the iterative form of the proximity gradient algorithm at the current point *A*_*k*_ is as follows:

$$A_{k+1}={prox}_{g/L_{f}}\left(G_{k}\right).$$

Then, we obtain pseudocode for the subproblems of solving *A*, *E*, *C* with fixed *B*, *R*, as shown in Algorithm 1.

Algorithm 1 The Algorithm of solution for *A*, *E*, *C* with fixed *B*, *R*

fixing {*B*, *R*}
while *not converged* do
 
${\left[E\right]}_{\Phi }={\left[S_{\lambda \mu^{-1}}\left(X-A+\mu^{-1}\Lambda \right)\right]}_{\Phi }$

 
${\left[E\right]}_{\Phi^{C}}={\left[X-A+\Lambda \right]}_{\Phi^{C}}$

 while *not converged* do
  
$L=\mathrm{diag}\left(B1_{K}\right)-B,\;\Gamma =\mathrm{diag}\left(1^{T}R\right)$

  
$S=R{\left(\frac{\theta }{\gamma }L+\Gamma \right)}^{-1/2}$

  
$L_{f}=\|\left(\mu +2\gamma \right)I-2\gamma SS^{T}\|$

  
$G=A-\frac{1}{L_{f}}\left(-\Lambda +\mu \left(A-\left(X-E\right)\right)+2\gamma \left(I-SS^{T}\right)A\right)$

  
$A={prox}_{g/L_{f}}\left(G\right)$ ,
  
$\text{where}\;{prox}_{g/L_{f}}\left(G\right)=U\Sigma_{g/L_{f}}V^{T},\;\Sigma_{g/L_{f}}=\mathrm{diag}\left({\left\{\sigma_{i}-\frac{1}{L_{f}}\right\}}_{+}\right),{\left\{\cdot \right\}}_{+}=\max\left\{0,\cdot \right\}$

 
$C={\left(\frac{\theta }{\gamma }L+\Gamma \right)}^{-1}R^{T}A$

• Fix {*A*, *C*, *E*} and update {*B*, *R*}

Given {*A*, *C*, *E*}, Problem Eq. 10 with respect to *B* and *R* is a jointly convex optimization problem, which can be solved independently.

Updating *R*.

When fixing {*A*, *E*, *C*}, the optimization function related to *R* can be written as follows:

$$L^{\prime }\left(r_{i},\alpha \right)=\underset{k}{\sum }r_{i,k}\left(\|A_{i}-C_{k}{\|}^{2}+\sigma \log\left(r_{i,k}\right)\right)+\alpha \left(\underset{k}{\sum }r_{i,k}-1\right).$$ (20)

The KKT condition is ‖*A*_*i*_ − *C*_*k*_‖^2^ + *σ*(1 + log (*r*_*i*,*k*_)) + *α* = 0 and *∑*_*k*_ *r*_*i*,*k*_ = 1, *r*_*i*,*k*_ ≥ 0, *∀k* ∈ {1, …, *K*}. Then we have the analytic solution of *R* given by *r*_*i*,*k*_ = exp (‖*A*_*i*_ − *C*_*k*_‖^2^/*σ* − (1 + *α*/*σ*)). Owing to *∑*_*k*_ *r*_*i*,*k*_ = 1, we can get $\text{exp}\left(1+\alpha /\sigma \right)=\sum_{k=1}^{K}\text{exp}\left(-\|A_{i}-C_{k}{\|}^{2}/\sigma \right)$ . Then we can rewrite *r*_*i*,*k*_ as

$$r_{i,k}=\frac{\text{exp}\left(-\|A_{i}-C_{k}{\|}^{2}/\sigma \right)}{\sum_{k=1}^{K}\text{exp}\left(-\|A_{i}-C_{k}{\|}^{2}/\sigma \right)}.$$ (21)

Updating *B*.

The term associated with *B* in optimization function (10) is to find the minimum spanning tree among cluster centers in *C*, which can be solved *via* Kruskal’s algorithm (Kruskal, 1956; Cormen et al., 2022). Then we can sort out the pseudocode for the subproblem of solving *B*, *R* with fixed *A*, *C*, *E* as shown in Algorithm 2.

Algorithm 2 The Algorithm of solution for *B*, *R* with fixed *A*, *C*, *E*

fixing {*A*, *C*, *E*}
*d*_*k*,*k*′_ = ‖*C*_*k*_ − *C*_*k*′_‖^2^, *∀k*, *∀k*′
Obtain *B* by solving optimization Problem Eq. 9 *via* Kruskal’s algorithm
Compute *R* with each element as $r_{i,k}=\frac{\exp\left(-\|A_{i}-C_{k}{\|}^{2}/\sigma \right)}{\sum_{k=1}^{K}\exp\left(-\|A_{i}-C_{k}{\|}^{2}/\sigma \right)}$

Finally, combining the two subproblems above, we formulate the complete pseudocode of exact RobustTree algorithm in Algorithm 3. Fortunately, as it turns out, in the *k*th iteration of *B* and *R*, we do not have to solve the subproblem $\left(A_{k+1}^{*},E_{k+1}^{*},C_{k+1}^{*}\right)=L\left(A,C,E,B_{k},R_{k}\right)$ exactly, corresponding to line 8-17 of Algorithm 3. Rather, when solving this subproblem, updating *A*_*k*_, *C*_*k*_ and *E*_*k*_ once is sufficient for them to converge to the optimal solution of RobustTree problem. This leads to an inexact RobustTree algorithm (see Algorithm 4).

Algorithm 3 The exact RobustTree algorithm

Input: *X*, *λ*, *θ*, *γ*, and *σ*
Output: *A*, *C*, *E*, *B*, *R*
Initialize *A* by *ZF*(*X*), *K*, *C*
While *not converged* do
 *d*_*k*,*k*′_ = ‖*C*_*k*_ − *C*_*k*′_‖^2^, *∀k*, *∀k*′
 Obtain *B* by solving Problem Eq. 9 *via* Kruskal’s algorithm
 Compute *R* with each element as $r_{i,k}=\frac{\exp\left(-\|A_{i}-C_{k}{\|}^{2}/\sigma \right)}{\sum_{k=1}^{K}\exp\left(-\|A_{i}-C_{k}{\|}^{2}/\sigma \right)}$

 While *not converged* do
  
${\left[E\right]}_{\Phi }={\left[S_{\lambda \mu^{-1}}\left(X-A+\mu^{-1}\Lambda \right)\right]}_{\Phi }$

  
${\left[E\right]}_{\Phi^{C}}={\left[X-A+\Lambda \right]}_{\Phi^{C}}$

  While *not converged* do
   
$L=\mathrm{diag}\left(B1_{K}\right)-B,\;\Gamma =\mathrm{diag}\left(1^{T}R\right)$

   
$S=R{\left(\frac{\theta }{\gamma }L+\Gamma \right)}^{-1/2}$

   
$L_{f}=\|\left(\mu +2\gamma \right)I-2\gamma SS^{T}\|$

   
$G=A-\frac{1}{L_{f}}\left(-\Lambda +\mu \left(A-\left(X-E\right)\right)+2\gamma \left(I-SS^{T}\right)A\right)$

   
$A={prox}_{g/L_{f}}\left(G\right)$ ,
   
$\text{where}\;{prox}_{g/L_{f}}\left(G\right)=U\Sigma_{g/L_{f}}V^{T},\;\Sigma_{g/L_{f}}=\mathrm{diag}\left({\left\{\sigma_{i}-\frac{1}{L_{f}}\right\}}_{+}\right),{\left\{\cdot \right\}}_{+}=\max\left\{0,\cdot \right\}$

$C={\left(\frac{\theta }{\gamma }L+\Gamma \right)}^{-1}R^{T}A$

Algorithm 4 The inexact RobustTree algorithm

Input: *X*, *λ*, *θ*, *γ*, and *σ*
Output: *A*, *C*, *E*, *B*, *R*
Initialize *A* by *ZF*(*X*), *K*, *C*
while *not converged* do
 *d*_*k*,*k*′_ = ‖*C*_*k*_ − *C*_*k*′_‖^2^, *∀k*, *∀k*′
 Obtain *B* by solving Eq. 9 *via* Kruskal’s algorithm
 Compute *R* with each element as $r_{i,k}=\frac{\exp\left(-\|A_{i}-C_{k}{\|}^{2}/\sigma \right)}{\sum_{k=1}^{K}\exp\left(-\|A_{i}-C_{k}{\|}^{2}/\sigma \right)}$

 
${\left[E\right]}_{\Phi }={\left[S_{\lambda \mu^{-1}}\left(X-A+\mu^{-1}\Lambda \right)\right]}_{\Phi }$

 
${\left[E\right]}_{\Phi^{C}}={\left[X-A+\Lambda \right]}_{\Phi^{C}}$

 
$L=\mathrm{diag}\left(B1_{K}\right)-B,\;\Gamma =\mathrm{diag}\left(1^{T}R\right)$

 
$S=R{\left(\frac{\theta }{\gamma }L+\Gamma \right)}^{-1/2}$

 
$L_{f}=\|\left(\mu +2\gamma \right)I-2\gamma SS^{T}\|$

 
$G=A-\frac{1}{L_{f}}\left(-\Lambda +\mu \left(A-\left(X-E\right)\right)+2\gamma \left(I-SS^{T}\right)A\right)$

 
$A={prox}_{g/L_{f}}\left(G\right)$ ,
 
$\text{where}\;{prox}_{g/L_{f}}\left(G\right)=U\Sigma_{g/L_{f}}V^{T},\;\Sigma_{g/L_{f}}=\mathrm{diag}\left({\left\{\sigma_{i}-\frac{1}{L_{f}}\right\}}_{+}\right),{\left\{\cdot \right\}}_{+}=\max\left\{0,\cdot \right\}$

 
$C={\left(\frac{\theta }{\gamma }L+\Gamma \right)}^{-1}R^{T}A$

#### 2.4.3 Convergence analysis

Since optimization Problem Eq. 10 is non-convex, many local optimal solutions are possible. We perform theoretical convergence analysis as shown in Theorem 1.

Theorem 1. Let {*B*_*l*_, *R*_*l*_, *A*_*l*_, *C*_*l*_, *E*_*l*_} *be the solution of Problem* (10) *in the* *l*th *iteration, and let* $L_{l}=L\left(B_{l},R_{l},A_{l},C_{l},E_{l}\right)$ *be the corresponding objective function value; then we have:*

1. $\left\{L_{l}\right\}$ *monotonically decreasing and*
2. *Sequences* {*B*_*l*_, *R*_*l*_, *A*_*l*_, *C*_*l*_, *E*_*l*_} *and* $\left\{L_{l}\right\}$ *converging.*

Proof. Let {*B*_*l*_, *R*_*l*_, *A*_*l*_, *C*_*l*_, *E*_*l*_} be the solution obtained in the *l*th iteration. By Algorithm 3, at the (*l* + 1)th iteration, we have

$$\begin{array}{cc} L\left(B_{l},R_{l},A_{l},E_{l},C_{l}\right) & \geq L\left(B_{l+1},R_{l},A_{l},E_{l},C_{l}\right)\geq L\left(B_{l+1},R_{l+1},A_{l},E_{l},C_{l}\right)\\ & \geq L\left(B_{l+1},R_{l+1},A_{l+1},E_{l+1},C_{l}\right)\geq L\left(B_{l+1},R_{l+1},A_{l+1},E_{l+1},C_{l+1}\right). \end{array}$$

Then, sequence $\left\{L_{l}\right\}$ is monotonically decreasing. In addition, since L(B,R,A,C,E) is lower-bounded by −*γσM*log*K*, $L^{*}$ exists such that $\left\{L_{l}\right\}$ converges to $L^{*}$ according to the Monotonic Convergence Theorem. Then, we prove that sequence {*B*_*l*_, *R*_*l*_, *A*_*l*_, *C*_*l*_, *E*_*l*_} converges. Owing to the compactness of feasible sets *B* and *R*, the sequence {*B*_*l*_, *R*_*l*_} converges to {B*, R*} as *l* → *∞*. Based on the ADAL algorithm (Shang et al., 2014), {*A*_*l*_, *E*_*l*_} converges to {A*, E*}. Since $C={\left(\frac{\theta }{\gamma }L+\Gamma \right)}^{-1}R^{T}A$ , {*C*_*l*_} converges to $C^{*}={\left(\frac{\theta }{\gamma }L^{*}+\Gamma^{*}\right)}^{-1}R^{*T}A^{*}$ , where L* = diag (B*1)—B*, Γ* = diag (**1** ^*T*^ R*).

#### 2.4.4 Adaptive parameter selection

We denote missing rate as *s* in the observed input SCS data. Then we select the hyper-parameters in Problem Eq. 10 as follows:

$$\lambda =\frac{1+3s}{\sqrt{\max\left(M,N\right)}},\theta =\frac{\sqrt{M}}{N\sqrt{N}},\gamma =\frac{M}{N\sqrt{N}},\sigma =\frac{\text{var}\left(\text{X}\right)}{\max\left(M,N\right)},$$ (22)

where the selection of *λ* refers to Chen Z. et al. (2020). And we choose the other parameters to coordinate the value of each single item in Problem 10 with a similar magnitude during the optimization process.

We initialize *K* as following:

$$K=\left\{\begin{array}{ccc} M & & if\;M\leq 500;\\ \frac{M}{5} & & if\;500<M\leq 1000;\\ \frac{M}{50} & & if\;M>1000. \end{array}\right.$$ (23)

Each cell *i* is assigned to cluster *k*, which has the maximum value *r*_*i,j*_, *∀j* ∈ {1, …, *K*}, i.e., *k* = arg max_*j*∈{1,…,*K*}_ *r*_*i,j*_, and finally remove the repeated cluster centers to get the final cluster centers. With the above parameter settings, RobustTree can be applied to the reconstruction of continuous trajectory and discrete-state topological structure.

### 2.5 Evaluations

To evaluate cluster assignment and data recovery performance, we adopt the following measurements, including 1) adjusted rand index (ARI) (Rand, 1971; Qiu et al., 2017; Chen et al., 2019); 2) the error rate of the recovered matrixes to the ground truth (Chen Z. et al., 2020); 3) the percentage of missing entries imputed correctly (Miura et al., 2018; Chen Z. et al., 2020) and 4) the false positives and false negatives (FPs + FNs) ratios of output genotype matrix to input genotype matrix for scSNV data (Miura et al., 2018; Chen Z. et al., 2020).

## 3 Results

### 3.1 RobustTree reconstructs continuous trajectories on noisy simulation data with high accuracy

**FIGURE 1:**
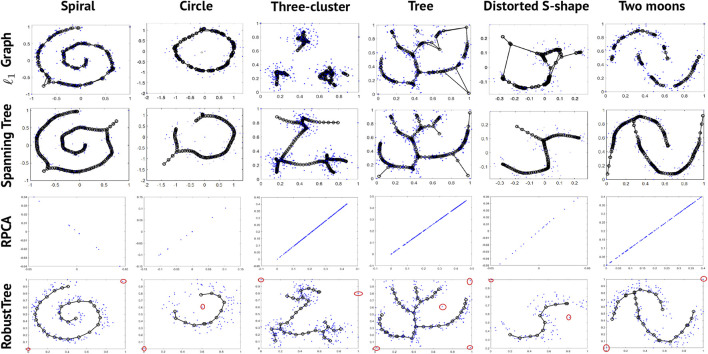
Results of 4 algorithms on 6 synthetic datasets with continuous topological structure. The red circle points in the fourth row are the artificially added noise points.

To demonstrate that RobustTree can preserve the global structure and handle high-noise data with continuous topology, we apply RobustTree to 6 simulated datasets with continuous trajectories. The original data are taken from Mao et al. (2016), which contain 200 (Spiral), 100 (circle), 300 (Three-cluster), 300 (Tree), 100 (Distorted S-shape), and 200 (Two moons) data points, respectively. To test the robustness of RobustTree to noise, we add 1 to 4 sharp noise points (points in the red circle in the fourth row of Figure 1) to each datum. We compare the results of RobustTree with *l*_1_ Graph and Spanning Tree, which are two algorithms performing principal graph and structure learning based on inverse graph embedding (Mao et al., 2016), as well as RPCA/RobustClone (Chen Z. et al., 2020).

Although *l*_1_ Graph and Spanning Tree show better effectiveness and stability than the Polygonal Line method (Kégl et al., 2000), SCMS (Ozertem and Erdogmus, 2011), and Mapper (Singh et al., 2007) algorithms in the results of Figure 5 in Mao et al. (2016), they are unstable to sharp noise. As shown in Figure 1, *l*_1_ Graph and Spanning Tree will generate redundant bifurcations or edges to connect noise points with parameters tuned for the original dataset by Mao et al. (2016), highlighting the obviously ineffective identification or removal of noise. When we directly apply RPCA, which is the first step in RobustClone algorithm, to this synthetic dataset, it tends to optimize the continuous topological structure into a straight line. This is probably due to the fact that the original two dimensional data matrices do not have the relatively low-rank property required by the RPCA method.

In contrast, since RobustTree optimizes denoising, clustering and tree reconstruction in a unified framework, it shows stronger robustness than other methods. As shown in Figure 1, RobustTree effectively extracts the sharp noise into *E*, clusters the recovered latent low-rank matrix, and reconstructs the intrinsic continuous trajectory with multiple types of data, including structures with linear or simple bifurcations.

### 3.2 RobustTree reconstructs continuous multi-branch trajectory effectively

**FIGURE 2:**
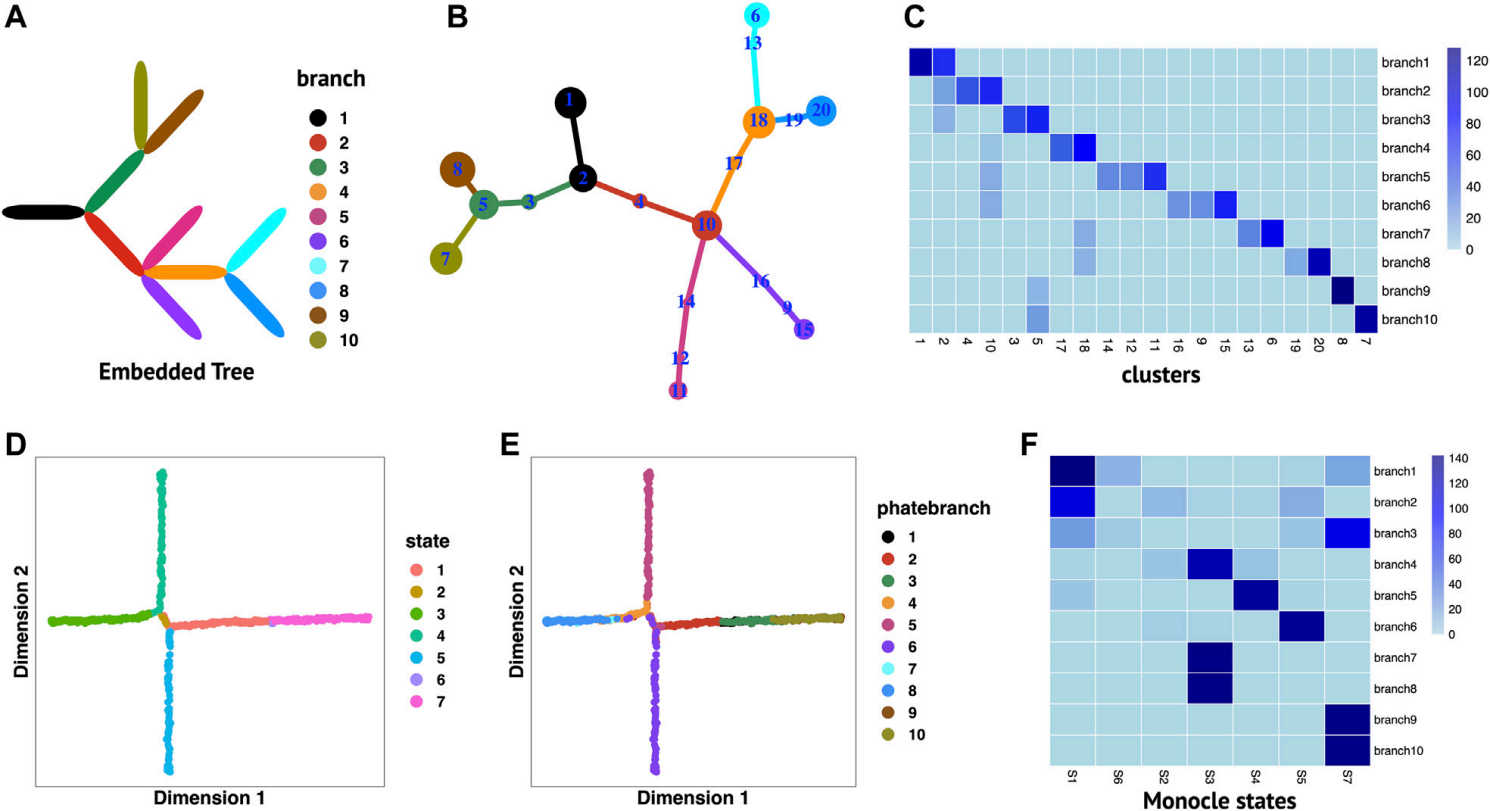
RobustTree reconstructs continuous multi-branch trajectory on PHATE data. **(A)** The real embedded tree structure of simulated PHATE data. **(B)** Tree trajectory reconstructed by RobustTree and visualized by R package igraph. The size of the cluster is proportional to the number of cells it contains, and the branch length is proportional to the distance between connected clusters. **(C)** Heatmap shows the percentage of cells in cluster (x-axis) distributed into real branch (y-axis). **(D)** Monocle2 reduces dimension on PHATE data. **(E)** Truth branch assignment on 2D embedding of Monocle2. **(F)** Heatmap shows the percentage of cells in Monocle states (x-axis) distributed into real branch (y-axis).

We perform RobustTree on simulated PHATE data to demonstrate its ability to handle data with continuous multi-branch development structures. The original data contain 1440 single cells and 60 genes (Moon et al., 2019), which imply an embedded continuous tree structure with 10 uniform branches to model a system where development along a given branch corresponds to increased expression of several genes (Figure 2A) (Chen et al., 2019; Moon et al., 2019).

RobustTree identifies 20 clusters and accurately reconstructs continuous trajectory with multi-branch on PHATE data, which contains three bifurcating events and one trifurcating event (Figure 2B). Figure 2C displays the distribution of clusters over branches. Clusters identified by RobustTree are almost exactly divided into a certain branch, except for clusters 2, 5, 10, and 18, which are located at branching points. The ARI between the branches identified by RobustTree and truth branch assignment is 0.6765. Since clusters at branching point contain cells from different branches, when excluding these clusters in ARI computation, we can achieve 0.9383.

We compare the RobustTree to Monocle2 (Qiu et al., 2017), a method that resolves complex single-cell trajectories using RGE, on this dataset. Monocle2 identifies 7 cell states, denoted as S1–S7, (Figures 2D, E, F), where real branches 1, 2 and 3, real branches 7, 8 and real branches 9, 10, are merged into S1, S3, and S7, respectively, leading to 6 main Monocle2 branches (Figures 2D, E, F). The ARI between the cell states identified by Monocle2 and the truth branch assignment is 0.4427.

### 3.3 RobustTree recovers discrete cell evolutionary history accurately on simulation data

Tumor evolution has been a subject with longstanding discussion (Nowell, 1976; Chen Z. et al., 2020). In tumors, cells form subpopulations (subclones) with nearly or completely identical genetic compositions, usually making the number of subclones much smaller than the number of cells or the number of mutational sites. In practice, the observed single-cell data are often incorporated with random noise caused by technical errors, including sequencing errors and dropout events. Accordingly, an important topic in tumor single-cell data analysis involves recovering subclonal genotypes and reconstructing the evolutionary history of subclones from corrupted data. In this study, we can also perform RobustTree on tumor single-cell DNA sequencing data to study the above problems.

**FIGURE 3:**
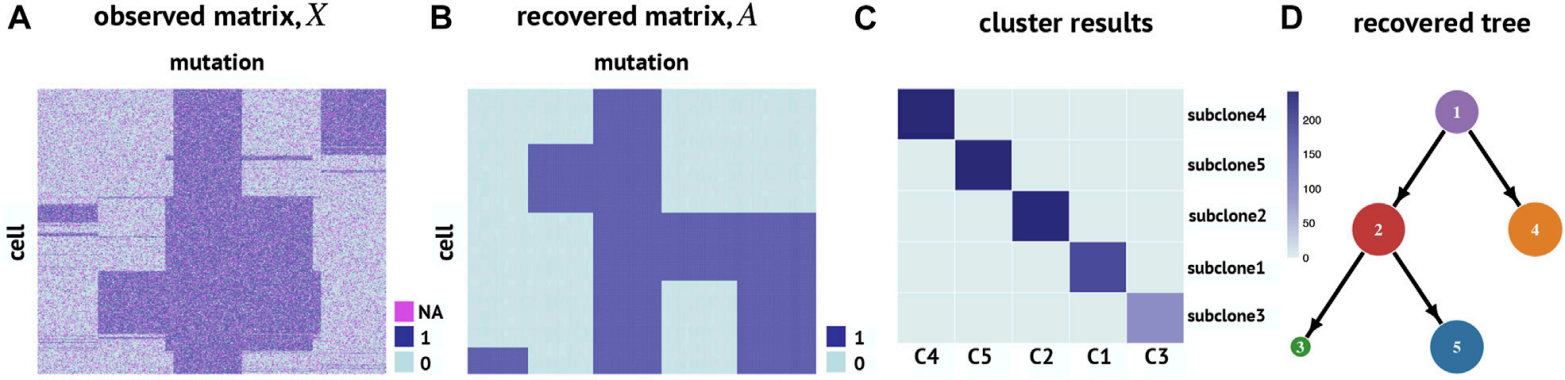
RobustTree reconstructs the tumor evolutionary tree on simulated single-cell DNA sequencing data. **(A)** Observed cell genotype matrix. **(B)** Recovered cell genotype matrix by RobustTree. **(C)** Heatmap shows the percentage of cells in each subclone (y-axis) distributed into real subclones (x-axis). **(D)** Subclone evolutionary tree reconstructed by RobustTree.

We first apply RobustTree to simulated scSNV data, which contain 1000 cells and 300 mutation sites, along with a sequencing error rate of 30% and a dropout rate of 20% (Chen Z. et al., 2020) (Figure 3A). There are 5 subclones along the real cell evolution tree, containing 193, 235, 93, 241, and 238 cells, respectively (Supplementary Figure S1). We use RobustTree to recover real cell genotypes and reconstruct the subclone evolutionary tree, and compare the performance of RobustTree to a state-of-the-art method, SCG, on the dataset.

RobustTree shows more accuracy than SCG on this simulation dataset. Specifically, for subclone identification, RobustTree identifies 5 subclone assignments on the tree, where the subclone assignment is exactly the same as the true subclone assignment (Figure 3C), that is, the ARI between the subclones identified by RobustTree and the true clone assignment is 1. However, SCG identifies 4 clusters, containing 278, 93, 391, and 238 cells, respectively. The ARI between the SCG clusters and the real clone assignment is 0.7245. For genotype recovery, RobustTree recovers the true genotype matrix with 100% accuracy (Figure 3B) with error rate and FPs + FNs(output/input) as 0, missing imputed correctly rate as 1. And the recovered tree structure (Figure 3D) exactly coincides with the real evolutionary history (Supplementary Figure S1). In contrast, the FPs + FNs(output/input) and missing imputed correctly rate are 0.2484, and 0.9603, respectively, leading to the total error rate of 3.77% in SCG results.

### 3.4 RobustTree recovers scSNV genotype and infers subclonal tree on high-grade serous ovarian cancer data

**FIGURE 4:**
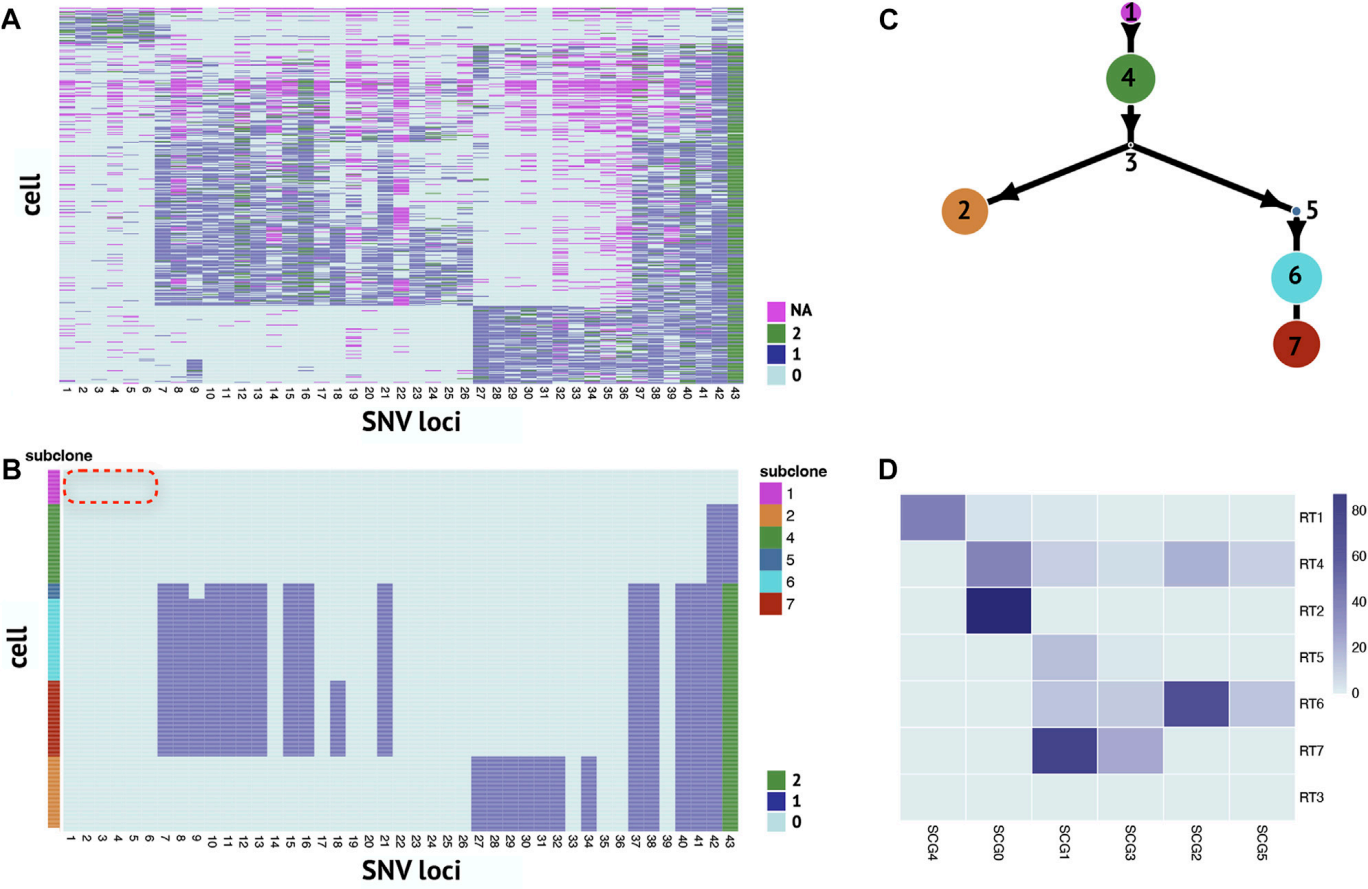
RobustTree reconstructs the tumor evolutionary tree on single-cell high-grade serous ovarian cancer data. **(A)** Observed noisy SNV genotype matrix. **(B)** Recovered SNV genotype matrix by RobustTree. **(C)** Subclone evolutionary tree reconstructed by RobustTree. **(D)** Heatmap shows the percentage of cells in each subclone (y-axis) distributed into SCG subclones (x-axis).

We apply RobustTree on the single-cell high-grade serous ovarian cancer (abbreviated as HGSOC) data (McPherson et al., 2016; Roth et al., 2016; Chen Z. et al., 2020), which contain 420 cells and 43 selected SNV sites with a missing rate of 10.7% (Figure 4A). RobustTree efficiently recovers the real genotype by imputing the missing data and correcting the noisy entries (Figure 4B) and identifies 7 subclones on the reconstructed MST, which contain 40, 87, 0, 92, 18, 95, and 88 cells, respectively (Figure 4C). Since subclone1 does not contain any mutations, it is assigned as the root subclone (Figure 4C).

Along the phylogenetic trees reconstructed along RobustTree, heterozygous mutations first occur at loci 42 and 43 in subclone4. Followed by homozygous mutation at locus 43 and heterozygous mutations at loci 37, 38, 40 and 41, all descendant subclones inherit these mutations. In addition, based on the observable ancestor subclone4, subclone2 accumulates mutations at loci 27-34, on the other branch, mutations mostly occur at loci 7–21.

We compare RobustTree to SCG (Roth et al., 2016) on this dataset. SCG identifies 6 clusters, where one main branch in SCG results, SCG0, contains cells from both subclone2 and subclone4 on RobustTree, and another main branch consisting of clusters SCG1, SCG3, SCG2, SCG5 corresponds to the branch comprised of subclones 5, 6, 7 on RobustTree. The cells of root cluster SCG4 dominate subclones1 of RobustTree, which can be interpreted as normal subclone (Figure 4D). However, SCG recovers some precancerous mutations in the normal cluster. In general, these precancerous mutations are expected to be carried in subsequent subclones, but they are completely absent in all progeny subclones (Figure 3 in Roth et al. (2016)). In contrast, RobustTree and RobustClone identify these mutations as false positive issues and recover the genotype without these mutations (Figure 4B), which seems more reasonable.

### 3.5 RobustTree recovers scCNA genotype on SA501X3F data

**FIGURE 5:**
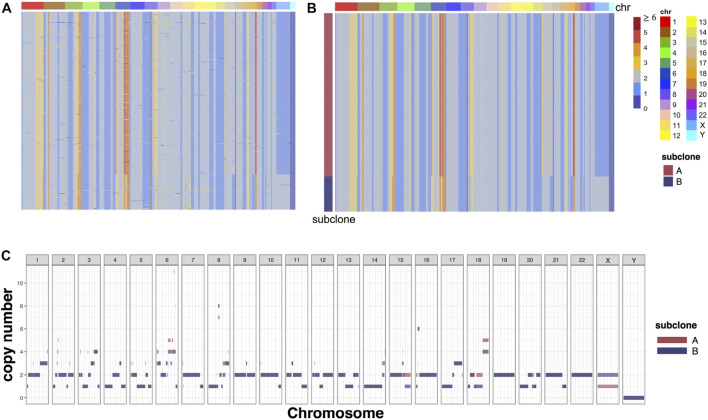
RobustTree reconstructs the tumor evolutionary tree on SA501X3F data. **(A)** Observed noisy CNA genotype matrix. **(B)** Recovered CNA genotype matrix by RobustTree. **(C)** Genotypes of subclones recovered by RobustTree.

RobustTree can also detect copy number heterogeneity and identify subclones in scCNA data. To demonstrate this, the RobustTree algorithm was applied to the cell copy number profile from primary triple-negative breast cancer (TNBC) xenograft passages, denoted as SA501X3F data (Zahn et al., 2017; Campbell et al., 2019). The data consist of the copy number states with 260 single cells and 20,651 genomic bins, as shown in Figure 5A. By leveraging data denoising, clustering, and tree structure reconstruction, RobustTree identifies two subclones (Figure 5B), containing 214 cells (subclone A) and 46 cells (subclone B), respectively. RobustTree recovers true cell genotypes (Figure 5B) and subclonal genotypes (Figure 5C), where the difference between the genotypes of the two subclones lies in the large fragment variation on the X chromosome, and small fragment variants on the chromosomes 6, 8, 15, and 18 (Figure 5BC).

This result is completely consistent with the result of RobustClone, that is, the ARI value between RobustTree and RobustClone classification is 1. And the cell assignment of subcloneA is also completely consistent with the major subclone identified in Zahn et al. (2017); Campbell et al. (2019). In the results of Zahn et al. (2017), there are two subclones derived from the subcloneB of RobustTree, which contain 28 and 18 cells, respectively. Since Zahn et al. (2017) identifies clones without explicitly correction for noise, there exists some uncertainty of assignment between these two minor subclones (Campbell et al., 2019). Therefore, classification results with two subclones are more robust (Chen Z. et al., 2020).

## 4 Conclusion

Computational methods based on SCS data to reconstruct inherent structure can provide important insight into the understanding of cell development and tumor progression. In this study, we propose a unified framework, RobustTree, which can recover corrupted entries and reconstruct the intrinsic structure underlying data. By coupling RPCA with tree structure optimization, RobustTree can leverage data denoising, clustering and tree structure reconstruction, as well as solve the tree optimization problem using adaptive parameter selections. By comparing to some other state-of-the-art methods, experimental results demonstrate the effectiveness of RobustTree on various types of datasets with different topological structures, including continuous cellular complex development trajectory and discrete cell evolutionary tree.

## Data Availability

Publicly available datasets were analyzed in this study. This data can be found here: https://liwang8.people.uic.edu/PAMI2016-PGSL.html; https://github.com/KrishnaswamyLab/PHATE; https://github.com/ucasdp/RobustClone; https://static-content.springer.com/esm/art%3A10.1038%2Fnmeth.3867/MediaObjects/41592_2016_BFnmeth3867_MOESM262_ESM.xls; https://zenodo.org/record/2363826#.Y_Wn1OzMKdY.
