# Supplementary material for: RobustTree: An adaptive, robust PCA algorithm for embedded tree structure recovery from single-cell sequencing data
Source: Front Genet. 2023 Mar 8;14:1110899. doi: 10.3389/fgene.2023.1110899 (PMC10030613; doi:10.3389/fgene.2023.1110899)
Supplement: Supplementary file 1 [file Image1.pdf]

Supplementary Material

1 SUPPLEMENTARY FIGURES

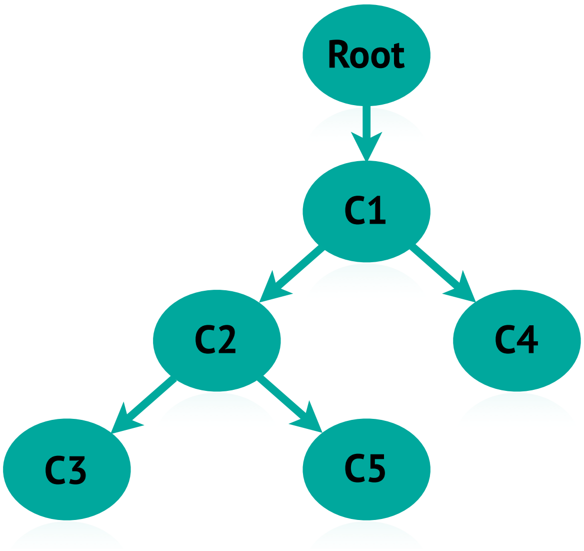

Figure S1. The real structure of the simulation study in Chen et al. (2020).

REFERENCES

Chen, Z., Gong, F., Wan, L., and Ma, L. (2020). RobustClone: a robust PCA method for tumor clone and evolution inference from single-cell sequencing data. *Bioinformatics* 36, 3299–3306
